# A simple method for assigning genomic grade to individual breast tumours

**DOI:** 10.1186/1471-2407-11-306

**Published:** 2011-07-21

**Authors:** Kristian Wennmalm, Jonas Bergh

**Affiliations:** 1 Department of Oncology and Pathology, Karolinska Institutet and University Hospital, Cancer Centrum Karolinska, R8:3, 171 76 Stockholm, Sweden; 2 Department of Oncology and Pathology, Karolinska Institutet and University Hospital, Radiumhemmet, 171 76 Stockholm, Sweden; 3 Medical Oncology Breast Unit, Paterson Institute for Cancer Research, Manchester, UK

**Keywords:** Breast Neoplasms, Microarray Analysis, Histology, Prognosis, Female

## Abstract

**Background:**

The prognostic value of grading in breast cancer can be increased with microarray technology, but proposed strategies are disadvantaged by the use of specific training data or parallel microscopic grading. Here, we investigate the performance of a method that uses no information outside the breast profile of interest.

**Results:**

In 251 profiled tumours we optimised a method that achieves grading by comparing rank means for genes predictive of high and low grade biology; a simpler method that allows for truly independent estimation of accuracy. Validation was carried out in 594 patients derived from several independent data sets. We found that accuracy was good: for low grade (G1) tumors 83- 94%, for high grade (G3) tumors 74- 100%. In keeping with aim of improved grading, two groups of intermediate grade (G2) cancers with significantly different outcome could be discriminated.

**Conclusion:**

This validates the concept of microarray-based grading in breast cancer, and provides a more practical method to achieve it. A simple R script for grading is available in an additional file. Clinical implementation could achieve better estimation of recurrence risk for 40 to 50% of breast cancer patients.

## Background

Decisions regarding medical treatment in early breast cancer are guided by disease stage, morphological characteristics (microscopic grading), and selected biological factors - notably expression of oestrogen and progesterone receptors and the *HER2 *oncogene [1]. The prognostic and therapy-predictive accuracies of these factors are limited, resulting in over- and under-treatment of patients. A decade of experience from large scale gene expression studies have underscored the relevance of some factors, as well as indicated potential improvements. Retrospective data reveal a high degree of concordance between microarray-technology and established methods for determining oestrogen and progesterone receptor status [2-5], as well as expression of the *HER2 *oncogene [6]. Concerning histopathological grading, two studies have indicated a potential for improvement. Here, the intermediate grade (G2) seems to lack a biological correlate. Both gene expression-wise and with regards to prognosis approximately two-thirds of G2-tumors are similar to the low grade group whereas about one third resemble the high grade group [7,8], which means that 40-50% of breast cancer patients currently get an unnecessarily imprecise estimate of their risk of recurrence.

For research as well as clinical purposes, we have been interested to use proposed methods for microarray-based grading. The more extensively investigated method [8] - now commercially available through MapQuant Dx - involves calculation of a summary score from genes with increased expression in G1 and G3 cancers, respectively (weighted -1 and +1). To allow use across different microarray platforms, scores are transformed with scale and offset terms, calculated utilizing known microscopic grading in data to be classified, in a leave-one-out cross-validation. It is unclear if the leave-one-out approach adequately deals with the obvious risk of over-fitting, and the need for parallel microscopic grading is not appealing, rendering the method unpractical and insufficiently validated. The methods proposed by Ivshina et al [7] - prediction analysis of microarrays (PAM) and statistically weighted syndromes (SWS) - require training of the algorithm in the same original data for true reproduction, which is cumbersome and may deter potential users. Also, data to be classified may have to be normalized to training data to maintain accuracy. To validate and simplify the concept of microarray-based grading, we tested a method that only uses information contained in the single tumour profile of interest.

## Implementation

For Sotiriou's et. al. [8] set of 128 probe sets, the ranks (1 for a probe set with lowest expression in a given tumour profile, n for a gene with highest; n = number of probe sets on the microarray platform) were averaged for probe sets with increased expression in G3-tumors (n = 112), and those with increased expression in G1-tumors (n = 16), respectively. The higher average determines the genomic grade (we use Sotiriou's and co-workers term to distinguish from histopathological grade). Expression of grade-related genes is thus only related to other genes internal to the profile, resulting in a method that can classify individual profiles with no further information. Insensitivity to data set composition, no requirement for training data, and unbiased estimation of accuracy are key advantages. Sensitivity to differences in average expression between probes is a potential limitation.

## Results

**Figure 1:**
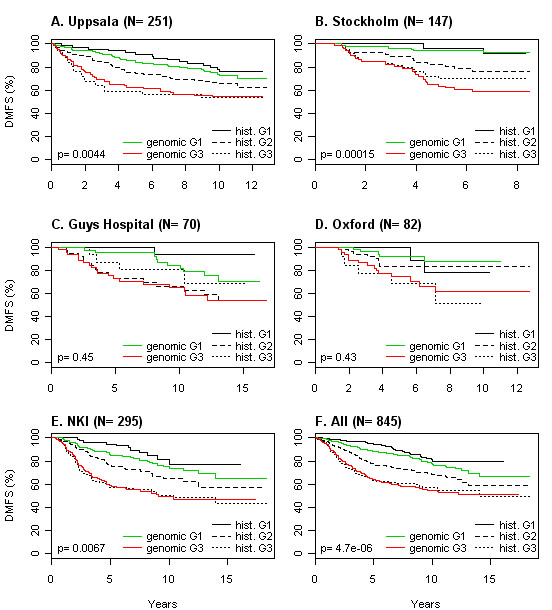
**Prognostic significance of microarray-based grading**. Tumours in the respective data sets graded with either the "genomic grade" (green- red) or by a pathologist (solid, dashed and dotted). Fraction of patients alive without distant metastasis (DMFS; y-axis), years (x-axis). Genomic grade determined with the optimized signature (reduced from 128 to 91 probe sets) and mean rank. P-values = log-rank test of difference for intermediate grade tumors (hist. G2; dashed line), when stratified by microarray-based grading. Insufficient statistical power in C and D: the number of events per groups small.

We tested the rank-based algorithm in a data set with 251 histopathologically graded breast cancers, here referred to as the Uppsala data set [9]. An assumption of equal average rank for G3-probe sets compared to G1-probe sets was not met: G1-probe sets had higher average expression than G3-probe sets. This resulted in bias towards low genomic grade classification calls; we therefore removed 37 lowly expressed genes from the larger group (G3-probe sets). With this optimization performed in the Uppsala cohort, we went on and validated the method in independent data (Table 1). Considerable accuracy was apparent in these published data sets - for the three series run on Affymetrix chip technology, 93%, 94%, and 83% of (histopathological) grade 1 tumours were correctly classified as low grade, and 85%, 100%, and 93% of (histopathological) grade 3 tumours were appropriately identified as high grade, translating to an overall accuracy of 88%, 97% and 88% for the Stockholm [10], Guys Hospital [11], and Oxford data [11]. The accuracy for the Netherlands Cancer Institute (NKI) data was somewhat inferior with an overall accuracy of 78%, possibly reflecting the fact that Agilent two-colour technology had been used [12]. The improvement in prognostic accuracy is illustrated in Figure 1: with array-based grading histologic G2 tumours (associated with intermediate outcome) are reclassified as either genomic G1 or G3 (associated with favourable or worse outcome, respectively). Further comparisons of original and simplified expression-based grading calls, microscopic grading calls, outcome, and unsupervised clustering results, can be found in Additional file 1.

**Table 1:** Agreement in classification between microarray-based and microscopic grading in five published data sets

		Histo-patological grade					
		
Cohort	Genomic grade	G1		G2		G3	
Uppsala	G1	**63**	**(93%)**	80	(62%)	4	(7.3%)
( N = 251)	G3	5	(7.4%)	48	(38%)	**51**	**(93%)**
Stockholm	G1	**26**	**(93%)**	44	(76%)	9	(15%)
( N = 147)	G3	2	(7.1%)	14	(24%)	**52**	**(85%)**
Guys Hospital	G1	**16**	**(94%)**	16	(43%)	0	
(N = 70)	G3	1	(5.9%)	21	(57%)	**16**	**(100%)**
Oxford	G1	**15**	**(83%)**	24	(48%)	1	(7.1%)
(N = 82)	G3	3	(17%)	26	(52%)	**13**	**(93%)**
NKI	G1	**64**	**(85%)**	68	(67%)	31	(26%)
(N = 295)	G3	11	(15%)	33	(33%)	**88**	**(74%)**

**In bold**: concordance in low and high grade (G1 and G3) assignments, describing the accuracy of microarray-based grading. Per cent per column and data set. Genomic grade determined with the optimized signature (reduced from 128 to 91 probe sets) and mean rank. NKI, the Netherlands Cancer Institute.

## Conclusions

Routine use of microarray or other RNA-profiling technology in early breast cancer is a likely future development, and projects to test this clinically are underway. Common to all these efforts is the aim to achieve improved prognostic and treatment predictive information. We take particular interest in microarray-based grading as it offers some key advantages over other proposed strategies (MammaPrint and Oncotype Dx etcetera): grade is well-known and interpretation in relation to clinical guidelines is straightforward. Change of practice - with the potential to spare patients from aggressive therapy - is likely to happen faster than with signatures introducing new taxonomies with unclear relationships to established prognostic and treatment predictive markers. Focusing on factors of known importance - like grade - also makes it possible to motivate microarray technology as an improvement in medical technology: similar or improved information (compared to that contributed by the pathology department) can be achieved at a competitive cost. Suggested methods to achieve this - in their current published form [7,8] - are disadvantaged by methodological complexity and uncertain independent accuracy, which has motivated us to design a simpler data set-independent method and evaluate it in several tumour series. This can hopefully increase interest in the concept of microarray-based grading; an R script for the necessary calculations is available in an additional file (Additional file 2).

## Availability and requirements

• Project name: expression grade.txt

• Project home page: available as an additional file (Additional file 2)

• Operating system(s): any setting where R works

• Programming language: R (http://www.r-project.org/)

• Other requirements: none

• License: GNU GPL (R)

• Any restrictions to use by non-academics: none

## Competing interests

The authors declare that they have no competing interests.

## Authors' contributions

KW wrote the R script, performed analyses, and drafted the manuscript. JB substantially contributed to data acquisition and manuscript writing. Both authors read and approved the final manuscript.

## Pre-publication history

The pre-publication history for this paper can be accessed here:

http://www.biomedcentral.com/1471-2407/11/306/prepub

## Supplementary Material

Additional file 1 **Additional analyses and methods**. comparisons of original expression-based grading calls and grade (Table S1), original and simplified calls (Table S2), outcome (Figure S1), unsupervised clustering results (Table S3), expanded materials and methods, and R script instructions. Click here for file

Additional file 2 **Expression grade.txt**. An R script for calculation of the simplified expression-based grade. Click here for file
