# Supplementary material for: A simple method for assigning genomic grade to individual breast tumours
Source: BMC Cancer. 2011 Jul 21;11:306. doi: 10.1186/1471-2407-11-306 (PMC3150343; doi:10.1186/1471-2407-11-306)
Supplement: Additional file 1 — Additional analyses and methods. comparisons of original expression-based grading calls and grade (Table S1), original and simplified calls (Table S2), outcome (Figure S1), unsupervised clustering results (Table S3), expanded materials and methods, and R script instructions. [file 1471-2407-11-306-S1.PDF]

Supplementary Table 1. Agreement in classification between the original Genomic Grade (Sotiriou et.al., J Natl Cancer Inst 2006) and microscopic grading in five published data sets.

		Histo-patological grade					
**Cohort**	**Genomic grade**	G1		G2		G3	
Uppsala	G1	**63**	**(93 %)**	92	(72 %)	6	(11 %)
(N = 251)	G3	5	(7.4 %)	36	(28 %)	**49**	**(89 %)**
Stockholm	G1	**26**	**(93 %)**	45	(78 %)	12	(20 %)
(N = 147)	G3	2	(7.1 %)	13	(22 %)	**49**	**(80 %)**
Guys Hospital	G1	**16**	**(94 %)**	18	(49 %)	0	
(N = 70)	G3	1	(5.9 %)	19	(51 %)	**16**	**(100 %)**
Oxford	G1	**16**	**(89 %)**	37	(74 %)	2	(14 %)
(N = 82)	G3	2	(11 %)	13	(26 %)	**12**	**(86 %)**
NKI	G1	**62**	**(83 %)**	59	(58 %)	23	(19 %)
(N = 295)	G3	13	(17 %)	42	(42 %)	**96**	**(81 %)**

In bold: concordance in low and high grade (G1 and G3) assignments, describing the accuracy of microarray-based grading. Per cent per column and data set. Genomic grade determined with the original signature (128 probe sets) and according to Sotiriou et.al., J Natl Cancer Inst 2006. NKI, the Netherlands Cancer Institute.

Supplementary Table 2. Agreement in classification between the original Genomic Grade (Sotiriou et.al., J Natl Cancer Inst 2006) and the simplified in five published data sets.

		Simplified approach			
**Cohort**	**Genomic grade**	G1		G3	
Uppsala	G1	**147**	**(99 %)**	16	(15 %)
(N = 253)	G3	2	(1.3 %)	**88**	**(85 %)**
Stockholm	G1	**85**	**(99 %)**	6	(8.2 %)
(N = 159)	G3	1	(1.2 %)	**67**	**(92 %)**
Guys Hospital	G1	**41**	**(100 %)**	3	(6.5 %)
(N = 87)	G3	0		**43**	**(93 %)**
Oxford	G1	**53**	**(100 %)**	15	(33 %)
(N = 99)	G3	0		**31**	**(67 %)**
NKI	G1	**142**	**(87 %)**	2	(1.5 %)
(N = 295)	G3	21	(13 %)	**130**	**(98 %)**

In bold: concordance in low and high grade (G1 and G3) assignments, describing the similarity between the original (Sotiriou et.al., J Natl Cancer Inst 2006; 128 probe sets) and the simplified approach (91 probe sets and rank means). Per cent per column and data set. NKI, the Netherlands Cancer Institute. The total number of assessed tumours is larger (893) than in Supplementary Table2, as tumours with missing (histologic) grade could also be considered.

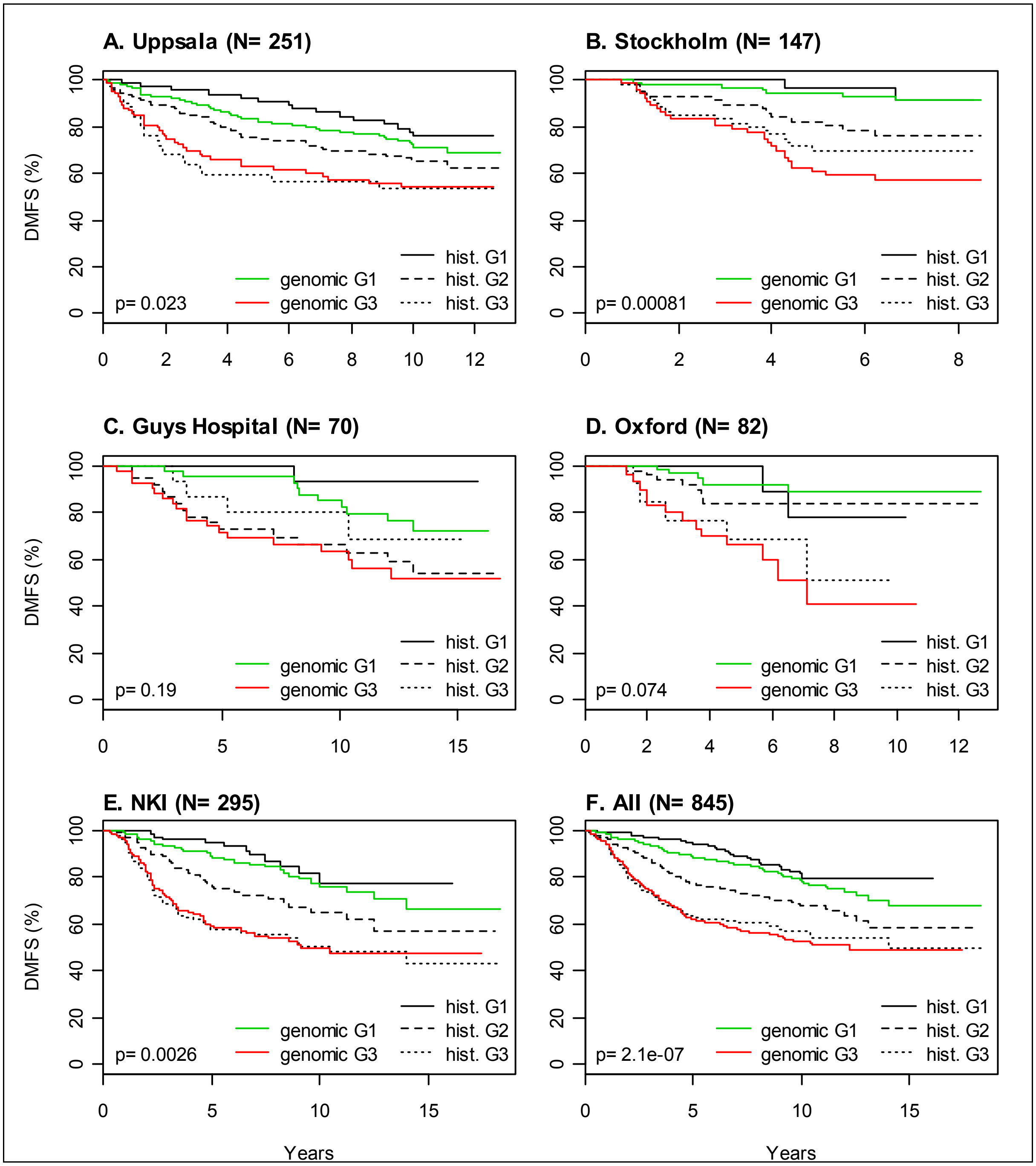

Supplementary Figure 1. Prognostic significance of the original Genomic Grade signature (green–red) vs. microscopic assessment (full, dashed, or dotted). Genomic grade determined with the full signature (Sotiriou et.al., J Natl Cancer Inst 2006; 128 probe sets). P-value = log-rank test for difference between genomic grade G1/G3 tumours of intermediate grade (histologic G2).

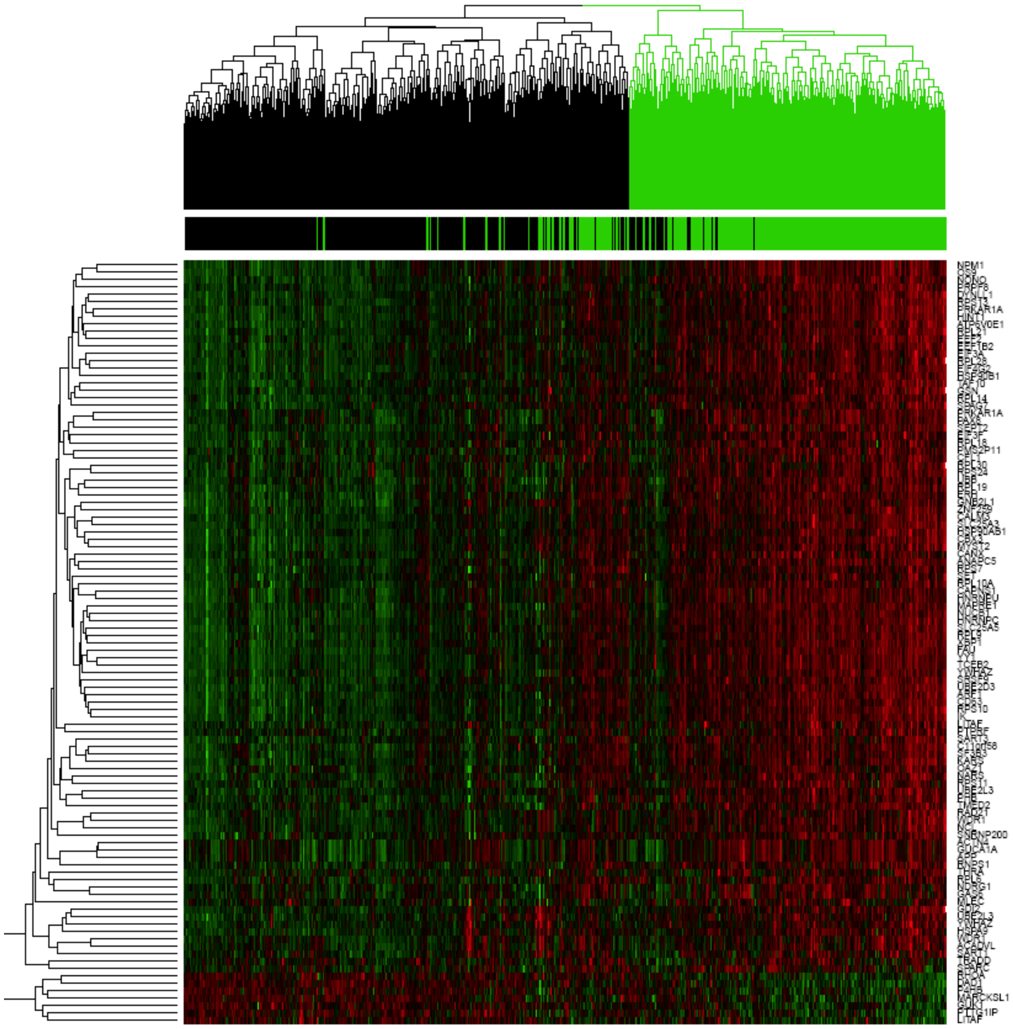

Supplementary Table 3. Agreement between unsupervised clustering and the simplified and original Genomic Grade (Sotiriou et.al., J Natl Cancer Inst 2006), respectively, in five merged data sets.

		Cluster	
**Signature**	**Genomic grade**	Black	Green
Simplified	G1	**439 (84 %)**	53 (14 %)
	G3	83 (16 %)	**318 (86 %)**
Original	G1	**441 (84 %)**	69 (19 %)
	G3	81 (16 %)	**302 (81 %)**

Hierarchical clustering with a Pearson correlation distance measure and complete linkage clustering. Tumours (n= 893) in columns and signature genes (103 common to all platforms) in rows. Red indicates high expression, green low. The top split in the dendrogram was used to define two clusters (black and green branches). Agreement with the simplified approach (91 probe sets and rank means; color bar on top in heatmap) and the original (Sotiriou et.al., J Natl Cancer Inst 2006; 128 probe sets) is shown.

Supplementary materials and methods

Data for the Stockholm and Uppsala tumour series are deposited at Gene Expression Omnibus (GEO; http://www.ncbi.nlm.nih.gov/geo/) with accession numbers GSE4922 and GSE1456. The Guys Hospital and Oxford data was downloaded from GEO (GSE6532); we used the normalized data contained in the 'Luminal.RData' file. For the Netherlands Cancer Institute (NKI) patients, data was downloaded from the authors' website. Information regarding distant metastasis-free survival and histopathological tumour grading was available for the Guys Hospital and Oxford data through GEO, and could be extracted for the NKI data from supplements to Fan et al.(1). For Stockholm, Uppsala, Guys Hospital and Oxford data, robust multichip average (RMA) normalization was used; for the NKI data the authors original preprocessing of the Agilent data was accepted, with multiple probes pertaining to individual genes (as defined by Entrez Gene identifiers) averaged. The microarray-based grade was achieved as follows: expression measures in an individual profile were ranked, ranging from 1 (for the most lowly expressed probe set) to n (for the most highly expressed, n= number of probe sets on the platform). For two sets of genes - predictive of low and high grade respectively - ranks were averaged, achieving a mean rank for the group of genes. If the mean rank for the low-grade genes was higher, this resulted in a low grade (genomic G1) assignment, if the opposite was true the profile was designated high grade (genomic G3). Sotiriou's et al.(2) signature was used initially, where 16 probe sets were predictive of low grade and 112 of high grade. In the training data (Uppsala data set), the mean rank was on average lower in the high grade probe sets, resulting in a bias towards low grade classification calls. The lowliest expressed G3-probe sets were removed until the average mean rank across the

Uppsala tumours was similar for G1 and G3 groups, resulting in a reduction from 112 to 75 G3-probe sets. This optimization of the algorithm was based entirely on Uppsala data, and validated in the other data sets.

R script

The deposited script is intended for use with the R language for statistical computing and graphics (http://cran.r-project.org/). A full description of handling of microarray data in R is beyond the scope of this document, only example code is supplied. Using the script involves four steps:

1. Import of data
2. Formatting data
3. Using the script
4. Exporting results

1. Import of data

Import of microarray data can be achieved in several ways, using standard functions in R such as read.table (e.g. for a tab-delimited text file) or more specialized functions like ReadAffy (in the affy R package intended for Affymetrix microarray data). Example code for affy:

```
> library(affy)
> setwd("K:/Documents/rankGGI/arraydata/")
```

Second line: set the working directory to where the raw data (CEL-files) are kept.

```
> theData <- ReadAffy()
> eset<-rma(theData)
```

This achieves normalization of data according to the RMA algorithm (Irrizarry Biostatistics 2003).

```
> dataMatrix <- exprs(eset)
```

This last line achieves a format compatible with the grading script. The format can be viewed by executing the following line, giving the first ten lines of dataMatrix:

```
> dataMatrix[1:10,]
```

	sample1.cel	sample2.cel	sample3.cel	sample4.cel
1007_s_at	12.102583	12.021306	10.919054	11.263000
1053_at	6.427339	6.735717	6.494163	6.402542
117_at	7.107009	7.785180	7.722527	7.294941
121_at	9.295500	9.373710	9.401608	9.509537
1255_g_at	4.214361	4.297733	4.358641	4.446044
1294_at	7.849894	8.396737	9.521005	8.587149
1316_at	6.090023	5.951153	6.284753	6.336231
1320_at	5.268741	5.415638	5.383881	5.359415
1405_i_at	5.248552	5.659654	7.295962	6.539422
1431_at	4.430049	4.417949	4.761806	4.485583

The gene expression data is represented as a table (or numerical matrix in R nomenclature) with individual samples as columns (e.g. tumours, here the arbitrary names “sample1-4,cel” were used) and probe sets (probing specific genes) as rows.

Values indicate expression in the corresponding tumour for specific probe sets. This table serves as an example of an appropriate format for the microarray data. Of note: it is in no way necessary to use R for normalization of Affymetrix data to use the grading script, importing a tab-delimited or other file with the microarray data into R is equivalent, as long as the right format is achieved (probe sets/genes in rows, tumour samples in columns).

2 Formatting data

As stated, the script is intended for use with a 'numerical matrix' containing the microarray data. Each row should contain expression values either for an Affymetrix probe set pertaining to the HG-U133A chip, or a specific gene indicated by an 'Entrez Gene ID', which is what the NCBI web site uses. When using Entrez Gene IDs, the user may want to average or by some other means collapse data in cases where several probes/probe sets in the data probe the same gene. Columns should contain tumour samples. It is also possible to enter a single expression profile as a numerical vector of expression values (for a single tumour sample).

The script also needs a description of the rows of the microarray data, as they appear in the numerical matrix in R. The row description should be a 'character vector', which can be achieved like this:

```
> rowdesc <- rownames(dataMatrix)
```

This line simply copies the rownames of 'dataMatrix' above (containing probe set identifiers for the Affymetrix U133A chip):

```
> rowdesc[1:10]
```

[1] "1007_s_at" "1053_at"   "117_at"   "121_at"   "1255_g_at" "1294_at"   "1316_at" "1320_at"   "1405_i_at" "1431_at"

IMPORTANT: the row descriptions can only be either one of two kids: an Affymetrix probe set pertaining to the HG-U133A chip, used for the original definition of the genomic grade signature (ref), or ‘entrez gene IDs’, for use with other microarray platforms.

3 Using the script

The ‘expression grade.txt’ text file contains the grading script. To use it, save a copy of the file in the directory where the CEL-files are kept (which is now your R working directory if you have used the sample code above). Execute:

```
> source("expression grade.txt")
> output <- rankclass(dataMatrix, rowdesc, annotation="u133a")
```

The second line runs the rankclass function defined in ‘expression grade.txt’. The arguments are:

‘dataMatrix’ = the microarray data imported in R, formatted as described.

‘rowdesc’ = the row descriptions formatted as described.

annotation = only the arguments “u133a” or “entrezgene” can be used, indicates what row descriptions you are using in the second argument (“rowdesc”).

The rankclass function returns the following:

```
> output
sample1.cel    sample2.cel    sample3.cel    sample4.cel
3              3              1              1
```

Output is a numerical vector containing the result of microarray-based grading, where 1= low grade, 3= high grade.

4 Exporting results

To export data into a text file execute the following:

```
> write.table(output, file="output.txt",col.names=F)
```

This produces a text file with tumour samples in the first column and grading in the second.

References

1. Fan C, Oh DS, Wessels L, et al. Concordance among gene-expression-based predictors for breast cancer. *N Engl J Med.* 2006;355(6):560-9.
2. Sotiriou C, Wirapati P, Loi S, et al. Gene expression profiling in breast cancer: understanding the molecular basis of histologic grade to improve prognosis. *J Natl Cancer Inst.* 2006;98(4):262-72.
